# Philadelphia Dental College

**Published:** 1882-05

**Authors:** 


### PHILADELPHIA DENTAL COLLEGE.

The nineteenth annual commencement.

The number of matriculates for the session was one hundred and thirty-eight.

The degree of D.D.S. was conferred on the following members of the class by the President of the Board of Trustees:

| NAME. | RESIDENCE. | NAME. | RESIDENCE. |
|---|---|---|---|
| Clarence Archer | West Indies. | R. Holt Jones | Delaware, |
| Thomas J. Burgess | Michigan. | A T Lamb | Maine. |
| Fred W. Cryderman | Ontario. | A. LaRosque | Canada. |
| Bernard Chedel | Georgia. | William W. Lazear | Illinois. |
| C. Byron Crandall | New York. | S. B. Luckie | Pennsylvania. |
| James R. Dickson | Canada. | Charles E. Mason | Ohio. |
| Walter A. Dorland | Canada. | F. H. McIntosh | Illinois. |
| George C. Duncan | New York. | C. A. Merrill | Georgia. |
| F. M. Edwards | New York. | J. B. Miesse | Pennsylvania. |
| Charles E. Faxon | Rhode Island. | P. J. Navarro | South America. |
| Otto Fenthol | Germany. | G. C. Nixon | Ohio. |
| Samuel J. Fisher | New York. | William F. Parks | Pennsylvania. |
| George A. Fowler | Pennsylvania. | William Peper | Pennsylvania. |
| Ernest W. Fox | England. | F. M. Poulson | Michigan. |
| J. S. Garner | South Carolina. | H. C. Read | New York. |
| C. H. Guthapfel | Pennsylvania. | Adolf Rettisch | New York. |
| William L. Haskell | Maine | F. M. Reynolds | Pennsylvania. |
| A. B. Harrower | Rhode Island. | George P. Rishel | New York. |
| J. A. Hartman | Pennsylvania. | E. Rodriguez | Cuba. |
| Leon F. Head | Pennsylvania. | A. D. Rosenthal | Pennsylvania. |
| Alfred Healey | New Jersey. | John H. Sailor | Indiana. |
| G. H. F. Herbst | Germany. | I. W. Scarborough, Jr. | Mississippi. |
| W. Hirschfeld | Germany. | B. F. Sleeper | Pennsylvania. |
| Thomas H. Holland | Pennsylvania. | P. L. Stoddard | New York. |
| C. J. O. Hotz | Switzerland. | A. E. Verrinder | California. |
| Charles R. Jefferis | Delaware. | Guy B. Vroom | Connecticut. |

